# Characteristics of patients initiating raloxifene compared to those initiating bisphosphonates

**DOI:** 10.1186/1472-6874-8-24

**Published:** 2008-12-23

**Authors:** Shonda A Foster, Kathleen A Foley, Eric S Meadows, Joseph A Johnston, Sara Wang, Gerhardt M Pohl, Stacey R Long

**Affiliations:** 1 US Outcomes Research, Eli Lilly and Company, Indianapolis, IN, USA; 2 Outcomes Research, Thomson Healthcare, Ann Arbor, MI, USA

## Abstract

**Background:**

Both raloxifene and bisphosphonates are indicated for the prevention and treatment of postmenopausal osteoporosis, however these medications have different efficacy and safety profiles. It is plausible that physicians would prescribe these agents to optimize the benefit/risk profile for individual patients. The objective of this study was to compare demographic and clinical characteristics of patients initiating raloxifene with those of patients initiating bisphosphonates for the prevention and treatment of osteoporosis.

**Methods:**

This study was conducted using a retrospective cohort design. Female beneficiaries (45 years and older) with at least one claim for raloxifene or a bisphosphonate in 2003 through 2005 and continuous enrollment in the previous 12 months and subsequent 6 months were identified using a collection of large national commercial, Medicare supplemental, and Medicaid administrative claims databases (MarketScan^®^). Patients were divided into two cohorts, a combined commercial/Medicare cohort and a Medicaid cohort. Within each cohort, characteristics (demographic, clinical, and resource utilization) of patients initiating raloxifene were compared to those of patients initiating bisphosphonate therapy. Group comparisons were made using chi-square tests for proportions of categorical measures and Wilcoxon rank-sum tests for continuous variables. Logistic regression was used to simultaneously examine factors independently associated with initiation of raloxifene versus a bisphosphonate.

**Results:**

Within both the commercial/Medicare and Medicaid cohorts, raloxifene patients were younger, had fewer comorbid conditions, and fewer pre-existing fractures than bisphosphonate patients. Raloxifene patients in both cohorts were less likely to have had a bone mineral density (BMD) screening in the previous year than were bisphosphonate patients, and were also more likely to have used estrogen or estrogen/progestin therapy in the previous 12 months. These differences remained statistically significant in the multivariate model.

**Conclusion:**

In this sample of patients enrolled in commercial, Medicare, and Medicaid plans, patients who initiated raloxifene treatment differed from those initiating bisphosphonates. Raloxifene patients were younger, had better overall health status and appeared to be less likely to have risk factors for new osteoporotic fractures than bisphosphonate patients. Differences in the clinical profiles of these agents may impact prescribing decisions. Investigators using observational data to make comparisons of treatment outcomes associated with these medications should take these important differences in patient characteristics into consideration.

## Background

Osteoporosis is the most common bone disease in the United States, affecting approximately 10 million people over the age of 50 [1]. An additional 18 million individuals have osteopenia, a precursor of osteoporosis [2]. Osteoporosis contributes to more than 1.5 million fractures each year and is the primary underlying cause of fractures in the elderly [3].

Both raloxifene and bisphosphonates are indicated for the prevention and treatment of postmenopausal osteoporosis. Raloxifene increases vertebral BMD and reduces the risk of vertebral fractures in postmenopausal women with and without preexisting vertebral fractures [4,5] but has not been shown to reduce the risk of nonvertebral fractures in patients versus placebo. However, studies of the efficacy of raloxifene in reducing the incidence of nonvertebral fractures were not adequately powered to determine whether this agent might also reduce the incidence of less frequent nonvertebral fractures [4]. Bisphosphonates reduce the risk of vertebral fractures, and two of the aminobisphosphonates (i.e., alendronate and risedronate) have been shown to reduce the risk of nonvertebral fractures [6,7].

The relative efficacy of raloxifene and bisphosphonates must be considered alongside the safety profiles associated with each treatment. The most common adverse events associated with raloxifene are hot flushes and leg cramps [8]. In addition, raloxifene has been associated with a two-fold increase in venous thromboembolism [9]. Data from clinical trials suggest that raloxifene reduces the risk of invasive breast cancer in postmenopausal women [9-12] and in 2007, raloxifene received approval from the FDA for reduction in risk of invasive breast cancer in postmenopausal women with osteoporosis and in postmenopausal women at high risk for invasive breast cancer [13]. The Raloxifene Use for the Heart (RUTH) trial, which was conducted with women at high risk of coronary events, indicated that there was no difference in overall mortality, cardiovascular mortality, or overall number of strokes versus placebo, however there was an increased risk of fatal stroke [12]. Thus, the risk benefit profile of raloxifene should be considered in women at risk for stroke. The most common adverse events associated with bisphosphonates are GI-related and include esophageal ulceration, stricture, and bleeding [14]. Osteonecrosis of the jaw, generally associated with tooth extraction and/or local infection has been reported in patients taking bisphosphonates. Most reported cases of bisphosphonate-associated osteonecrosis have been in cancer patients treated with intravenous bisphosphonates, but some have occurred in patients with postmenopausal osteoporosis [15-18].

Given the differing efficacy and safety profiles exhibited by raloxifene and the bisphosphonates, it is plausible that physicians would target these agents in such a way as to optimize the corresponding benefit/risk profile for individual patients. To explore this hypothesis, we sought to describe and compare the demographic and clinical characteristics of patients who initiate raloxifene therapy relative to patients initiating treatment with bisphosphonates.

## Methods

This study was conducted using a retrospective cohort design. Data are from MarketScan^® ^claims databases from Thomson Reuters, which reflect the healthcare experience of 62.1 million cumulative lives covered by a variety of private health plans, as well as Medicare supplemental and Medicaid insurance. Individuals with Medicare coverage are Medicare-eligible retirees with employer-sponsored Medicare Supplemental plans. Service claims during the period January 2002 through June 2005 were assessed for the commercial- and Medicare-insured populations, which were combined for analysis. Due to limited data availability, only service claims between January 1, 2002 and December 30, 2004 for Medicaid enrollees were examined. These data sources contain the pooled healthcare experience of enrollees and include records of inpatient services, inpatient admissions, outpatient services, and prescription drug claims. The patient data used in this analysis have been de-identified in compliance with Health Insurance Portability and Accountability Act (HIPAA) regulations, and, therefore, the study is exempt from Institutional Review Board approval.

The study population consists of women aged 45 and older who newly initiated treatment with raloxifene or a bisphosphonate (i.e., alendronate, ibandronate, and risedronate) any time during the period January 2003 through December 2005 (June 2004 for Medicaid). The date of the first prescription claim for raloxifene or a bisphosphonate in these periods represents the index date for each patient. Because the focus of this study is on new users, patients with a prescription for any osteoporosis treatment during the pre-period were excluded from the study. Patients were excluded if they had either an ICD-9 code or osteoporosis medication and dose indicative of Paget's disease. Patients were also excluded if they did not have prescription coverage in the pre- and post periods, or the index prescription was coded with a days supply of 0 or of more than 180 days, presumably in error.

### Measures

Demographic variables were defined as of the date of the index prescription claim and include age, race (for Medicaid patients only), regional location, urban/rural residence, and health plan type (i.e., capitated versus non-capitated). Clinical characteristics and conditions thought to potentially influence treatment choice were examined using ICD-9-CM codes for diagnoses, CPT codes for procedures (e.g., bone mineral density [BMD] scans, mammograms) and NDC codes for prior medication use. All codes used to identify confounding conditions are provided in Appendix A [see Additional File 1].

Fractures in the pre-period were captured using Health Plan Employer Data and Information Set (HEDIS) criteria and are identified as either hip, vertebral, or other non-vertebral (i.e., fractures of the extremities or ribs) [19]. HEDIS measurement guidelines were also used to identify patients who received a bone mineral density (BMD) test in the pre-period.

The Charlson Comorbidity Index (CCI) was calculated in the pre-period as a proxy for overall health status [20]. Finally, the numbers of inpatient, emergency room, outpatient, and pharmaceutical claims were evaluated for the pre-period.

The specialty of the provider most closely associated with the index prescription was also captured using an indirect method given that provider identifiers are not available on the prescription claims. The provider was determined by examining all claims for "evaluation and management" office visits in the 60 days prior to the index date. Claims with an osteoporosis diagnosis were scanned first to find the claim most temporally proximal to the prescription. If no claim with an osteoporosis diagnosis was found, then all claims in the 60-day period were scanned to identify the one closest to the prescription date. Provider specialty was coded as specialist (including OB/Gyn), or other (including primary care).

### Analysis

Statistical tests of significance of differences between groups were performed using chi-square tests for categorical measures and Wilcoxon rank-sum tests for continuous variables. Logistic regression was used to simultaneously examine the factors independently associated with raloxifene use as opposed to bisphosphonate use. Adjusted odds ratios and 95% confidence intervals are presented for all model covariates. All data are shown separately for individuals with commercial/Medicare insurance and those with Medicaid.

## Results

Prescription claims data were searched to identify the first claim for raloxifene or a bisphosphonate between January 2003 and December 2004 for patients. In the commercial/Medicare data 102,750 people had a claim for raloxifene and 356,145 had a claim for a bisphosphonate. For those receiving Medicaid there were 42,770 patients with a raloxifene claim and 184,193 with a bisphosphonate claim. The exclusion criteria having the greatest impact on the sample size were the requirements of not having an osteoporosis treatment in the pre-period and having continuous eligibility for 18 months. The final sample sizes were 17,983 raloxifene and 79,891 bisphosphonate users in the commercial/Medicare cohort and 11,504 raloxifene and 59,881 bisphosphonate users in the Medicaid cohort. Sample sizes following the application of exclusion criteria are provided in Appendix B [see Additional File 2].

Columns two and three of Table 1 present the demographic characteristics of the combined commercial/Medicare cohort. On average, patients initiating raloxifene were younger than those initiating bisphosphonate treatment (mean age = 62.2 and 64.2, respectively). Variation in the location of residence was also observed between raloxifene and bisphosphonate initiators, with higher proportions of raloxifene users residing in the South and in non-urban areas relative to bisphosphonate users. Finally, patients initiating raloxifene were more likely to have seen a specialist around the time of their index prescription than were patients initiating treatment with a bisphosphonate.

**Table 1:** Demographic characteristics (Commercial/Medicare and Medicaid)

	**Commercial/Medicare**		**Medicaid**	
	**Raloxifene**	**Bisphosphonates**	**Raloxifene**	**Bisphosphonates**
	**(N = 17,983)**	**(N = 79,891)**	**(N = 11,504)**	**(N = 59,881)**
Age (Mean, std.)*	62.2 (9.0)	64.2 (10.7)	69.3 (11.1)	71.8 (10.8)
				
Age group*	**%**	**%**	%	%
45–64	69.0	59.8	32.3	23.7
65–74	19.5*	20.2*	34.2*	34.0*
75 and over	11.5	20.0	33.5	42.2
				
Race *				
White	---	---	33.9	39.9
African American	---	---	5.3	6.2
Hispanic/Latino	---	---	15.1	17.2
Asian	---	---	29.6	21.4
Other	---	---	16.1	15.3
				
Medicare*	26.6	36.7	---	---
				
Location*				
North East	7.3	10.7	---	---
North Central	34.0	32.9	---	---
South	46.1	38.5	---	---
West	12.2	17.7	---	---
Unknown	0.4	0.3	---	---
				
Urban residence*	72.1	78.2	89.4	90.7
				
Insurance type*^1^				
Indemnity	34.5	39.3	82.3	82.3
PPO	44.7	40.8	---	---
Other	20.8	19.9	17.7	17.7
				
Provider specialty*				
Specialist^2^	18.8	16.8	6.5	4.9

*Test for differences between raloxifene and bisphosphonates statistically significant at p < 0.0001

^1 ^Difference not statistically different (p < 0.05) for Medicaid comparison

^2 ^Specialist versus primary care or other

Demographic characteristics of the Medicaid population are summarized in columns four and five of Table 1. As with the commercial/Medicare cohort, raloxifene users in the Medicaid population were significantly younger than their counterparts initiating bisphosphonate treatment. Variation in the racial/ethnic composition of the cohorts was also observed. Specifically, a greater proportion of raloxifene patients were Asian compared with bisphosphonate patients, whereas higher proportions of bisphosphonate patients were white, Hispanic, or African American. As with the commercial/Medicare population, raloxifene users with Medicaid were more likely to have seen a specialty provider than those using a bisphosphonate.

Table 2 displays the pre-period clinical characteristics of patients initiating raloxifene and bisphosphonates in both the commercial/Medicare and Medicaid cohorts. Patients initiating bisphosphonate treatment had slightly higher CCI scores and were more likely to have had a pre-period fracture than patients initiating raloxifene. Furthermore, raloxifene users less frequently had a BMD test or mammogram in the 12-month pre-period as compared to patients initiating bisphosphonate treatment. A much smaller proportion of Medicaid patients had a BMD screen relative to patients in the commercial/Medicare cohort.

**Table 2:** Pre-period clinical characteristics (Commercial/Medicare, and Medicaid)

	**Commercial/Medicare**		**Medicaid**	
	**Raloxifene**	**Bisphosphonates**	**Raloxifene**	**Bisphosphonates**
	**(N = 17,983)**	**(N = 79,891)**	**(N = 11,504)**	**(N = 59,881)**
Health status	Mean (STD)	Mean (STD)	Mean (STD)	Mean (STD)
CCI	0.5(1.1)*	0.7 (1.4)	0.9 (1.5)*	1.1 (1.6)
				
	%	%	%	%
Any fracture	10.0*	14.6	9.4*	14.2
Hip	0.4*	1.3	0.6*	1.3
Vertebral	1.4*	2.6	1.5*	2.4
Non-vertebral	9.2*	13.3	8.5*	12.7
				
Screening				
BMD test	43.2*	63.8	20.9*	27.6
Mammogram	8.4*	10.6	8.7	9.2
				
Confounding conditions				
Breast cancer	3.5*	5.5	6.0*	6.9
Endocrine disease	13.9	13.8	20.3	20.8
HIV	0.0	0.0	0.1	0.1
Liver disease	1.9	1.9	3.6	3.2
Bone cancer	0.0††	0.1	0.0	0.0
Other cancer	20.1	21.5	10.3	10.6
Alcoholism	0.1	0.1	0.1	0.1
Osteodystrophy	0.0	0.1	0.1	0.1
Nephritis	0.8*	1.1	1.9†	2.4
Rheumatoid arthritis	1.5*	2.7	2.4*	3.7
Any cardiovascular disease	5.9*	8.1	13.1	13.1
Thyroid disease	11.5*	12.6	8.3	7.9
Metabolic disorders	30.9†	29.7	25.5*	23.4
Osteoporosis	2.9*	6.9	5.3*	8.0
				
DVT/PE	0.0*	0.1	0.2†	0.4
Gastric ulcer	0.1	0.1	0.3	0.3
Peptic ulcer	0.1	0.0	0.6	0.5
Dysphagia	0.2	0.3	0.6	0.6
Gastroesophageal reflux	0.9	1.0	2.4	2.1
Gastritis	0.3	0.4	2.9*	2.2
				
Medications				
Glucocorticoids	13.4*	16.4	13.3*	16.6
Estrogen/HRT	38.4*	27.9	21.9*	13.7
Hormone deprivation therapy	1.2*	2.9	0.7*	1.6
Anticonvulsants	2.4*	3.1	5.4	5.8
Immunosuppressants	1.4*	2.6	1.1*	2.2
				
Resource utilization	Mean (STD)	Mean (STD)	Mean (STD)	Mean (STD)
Inpatient claims	0.1 (0.4)*	0.2 (0.5)	0.1 (0.5)*	0.1 (0.6)
ER claims	0.5 (2.0)*	0.6 (2.5)	2.1 (7.1)*	2.4 (8.4)
Outpatient claims	31.1 (32.7)*	36.4 (41.9)	49.0 (65.6)*	51.7 (70.3)
Rx claims	24.5 (24.9)*	26.2 (27.0)	46.9 (40.8)	49.9 (40.6)

Comparisons of raloxifene to bisphosphonates are statistically significant at: *p < 0.0001; †p < 0.001; †† p < 0.01

Of the comorbid conditions evaluated, the most prevalent among both raloxifene and bisphosphonate patients in the commercial/Medicare cohort were metabolic disorders, cancer other than breast or bone, and endocrine disease. Patients who initiated raloxifene within this insurance group were less likely to have breast cancers, nephritis, rheumatoid arthritis, thyroid disease, osteoporosis, metabolic disorders and cardiovascular disease than those who initiated bisphosphonate treatment. The most prevalent comorbid conditions among patients in both treatment groups within the Medicaid cohort were metabolic disorders, endocrine disease, and cardiovascular disease. Among the Medicaid patients, those who initiated raloxifene were less likely to have breast cancer, nephritis, osteoporosis and rheumatoid arthritis, but more likely to have metabolic disorders than Medicaid patients initiating a bisphosphonate. Gastrointestinal disorders affected less than 1% of patients in all cohorts with the exception of gastroesophageal reflux and gastritis, which were noted in 2–3% of Medicaid recipients on either a bisphosphonate or raloxifene.

In general, in both the commercial/Medicare and Medicaid groups, patients who initiated bisphosphonates were more likely to be taking a medication associated with bone complications than patients who initiated raloxifene. Approximately 13% of patients initiating therapy with raloxifene in the commercial/Medicare and Medicaid cohorts had a prescription for a glucocorticoid in the pre-period, compared to approximately 16% among patients initiating treatment with a bisphosphonate. However, a greater proportion of patients initiating raloxifene in the commercial/Medicare and Medicaid groups had previous or concurrent use of estrogen/hormone replacement therapy (HRT) within the past year compared to patients initiating bisphosphonate treatment. Raloxifene users in both insurance groups were less likely to have taken immunosuppressants or to have had hormone deprivation therapy.

With regard to resource utilization, patients initiating a bisphosphonate in both the commercial/Medicare and Medicaid cohorts had more inpatient, emergency room, outpatient, and prescription claims during the 12-month study period than patients initiating raloxifene.

Adjusted odds ratios for factors associated with use of raloxifene relative to a bisphosphonate are shown in Table 3. Among patients with commercial/Medicare insurance, those under age 65 and over age 75 were less likely to receive raloxifene relative to those between age 65 and 74. Among Medicaid recipients, however, the youngest cohort (age 45–64) was more likely than those age 65–74 to receive raloxifene. Non-white race/ethnicity was associated with a greater likelihood of receiving raloxifene among Medicaid recipients. For both insurance groups, seeing a specialty provider as opposed to a primary care or other physician was associated with increased odds of receiving raloxifene. Factors associated with a lower likelihood of receiving raloxifene and thus an increased likelihood of receiving a bisphosphonate include higher CCI, any prior fracture, pre-period BMD screening, a history of breast cancer (commercial/Medicare only) and DVT/PE and prior use of glucocorticoids. While a diagnosis of gastric ulcer in the pre-period increased the odds of receiving raloxifene among commercial/Medicare patients, it had no effect on whether Medicaid patients received raloxifene or a bisphosphonate, even though gastric ulcers were more common among Medicaid recipients (see Table 2). For both insurance groups, having received estrogen or other hormone replacement therapy in the pre-period was associated with greater odds of receiving raloxifene, however, glucocorticoid use in the pre-period was associated with greater odds of receiving bisphosphonates.

**Table 3:** Multivariate logistic regression results for characteristics associated with raloxifene vs. bisphosphonate use

	**Commercial/Medicare**		**Medicaid**	
	**Odds Ratio^1^**	**95% CI**	**Odds Ratio^1^**	**95% CI**
Age group				
45–64	0.881	0.810–0.957	1.509	1.431–1.592
65–74	REFERENCE	---	REFERENCE	---
75 and over	0.686	0.644–0.730	0.813	0.774–0.854
				
Race				
White	---	---	REFERENCE	---
African American	---	---	1.025	0.933–1.126
Hispanic/Latino	---	---	1.070	1.005–1.140
Asian	---	---	1.758	1.665–1.856
Other	---	---	1.299	1.220–1.382
				
Medicare	0.689	0.630–0.754	---	---
				
Location				
North East	0.672	0.629–0.718	---	---
North Central	0.935	0.898–0.973	---	---
South	REFERENCE	---	---	---
West	0.642	0.609–0.678	---	---
				
Urban residence	0.819	0.788–0.851	0.667	0.611–0.706
				
Insurance type				
Indemnity	0.986	0.935–1.040	1.128	1.068–1.192
PPO	1.031	0.985–1.079	---	---
Other	REFERENCE	---	REFERENCE	---
				
Provider specialty				
Primary care/Other	REFERENCE	---	REFERENCE	---
Specialist	1.108	1.060–1.158	1.264	1.160–1.377
				
Health status				
CCI	0.941	0.926–0.957	0.962	0.948–0.976
				
Fracture				
Hip	0.449	0.350–0.577	0.659	0.507–0.856
Vertebral	0.664	0.580–0.762	0.744	0.631–0.876
Non-vertebral	0.803	0.758–0.851	0.726	0.674–0.781
				
BMD test	0.412	0.398–0.426	0.664	0.628–0.703
Mammogram	0.968	0.909–1.031	0.953	0.886–1.026
				
Confounding conditions				
Breast cancer	0.746	0.679–0.819	1.021	0.928–1.123
DVT/PE	0.470	0.245–0.903	0.672	0.438–1.028
Gastric ulcer	2.273	1.181–4.375	0.861	0.585–1.267
Peptic ulcer	2.048	0.964–4.353	1.228	0.942–1.601
Dysphagia	0.974	0.694–1.367	1.200	0.917–1.571
Reflux	1.080	0.897–1.300	1.156	1.005–1.329
Gastritis	0.904	0.666–1.228	1.316	1.156–1.499
Osteoporosis	0.632	0.573–0.697	0.818	0.745–0.898
				
Medications				
Glucocorticoids	0.774	0.737–0.813	0.757	0.713–0.804
Estrogen/HRT	1.325	1.279–1.372	1.501	1.426–1.581

^1^Odds ratios that are less than 1.0 indicate that the covariate is associated with a lower likelihood of receiving raloxifene (or is associated with equivalently a higher likelihood of receiving a bisphosphonate). Odds ratios greater than 1.0 indicate that the covariate is associated with a higher likelihood of receiving raloxifene.

## Discussion

To date, our knowledge of the types of patients who initiate raloxifene for the prevention and treatment of osteoporosis has been limited. Using data from a large national commercial, Medicare, and Medicaid claims database, this study addresses this gap by describing the characteristics of patients who initiate raloxifene therapy. For comparison, we also report the characteristics of patients initiating therapy with a bisphosphonate.

The results of this study show demographic and clinical differences between patients in the two treatment groups. Patients initiating raloxifene therapy were younger and had a lower burden of comorbid illness than patients initiating bisphosphonate therapy. Within both the commercial/Medicare and Medicaid cohorts, patients initiating raloxifene were also less likely to have had a fracture or a BMD screen in the pre-period than patients initiating bisphosphonate therapy. These differences remained statistically significant in the multivariate model. Patients initiating raloxifene may be more likely to have been prescribed therapy for the prevention, rather than the treatment, of osteoporosis as suggested by their younger age relative to those using bisphosphonates as well as being less likely to have had a diagnosis of osteoporosis or fractures in the pre-period. Cadarette et al. reported that in a study population comprised of Medicare beneficiaries enrolled in statewide pharmaceutical benefit plans, raloxifene patients were younger and had a lower prevalence of fractures and comorbid conditions compared to patients on alendronate or risedronate [21]. Raloxifene patients also had a lower prevalence of diagnosed osteoporosis documented in Medicare claims than alendronate and risedronate recipients [21].

Prior or concomitant use of HRT was more common with raloxifene patients, which might imply that physicians consider raloxifene a treatment option when patients have or will be discontinuing HRT. Medicaid patients in both treatment groups had more claims of all types than patients in the commercial/Medicare cohort, however, a much smaller proportion of patients in the Medicaid cohort had a BMD test. This indicates a lack of osteoporosis screening which may result in underdiagnosis and undertreatment of osteoporosis within the Medicaid population. Differences in the racial/ethnic compositions of the treatment groups were also observed within the Medicaid population. A higher proportion of raloxifene users were Asian while higher proportions of whites, Hispanics, and African Americans initiated bisphosphonate treatment. Additional research should be conducted to explore these differences further, however, it may suggest differences in the cultural acceptance of SERM use or differences in perceptions of breast cancer risk which may result in these disparities.

Differences in the clinical characteristics of patients initiating raloxifene and bisphosphonates may be related to the comparative efficacy and safety profiles of the two treatments. For example, because the efficacy of raloxifene to reduce the risk of non-vertebral fractures has not been demonstrated in randomized, controlled trials, clinicians may be more likely to prescribe it for osteopenic patients or patients with less severe osteoporosis. Physicians may be less likely to prescribe raloxifene in older patients due to the increased risk of venous thromboembolism associated with its use. On the other hand, clinicians may be more inclined to use raloxifene in certain patients, for example younger women with a relatively low risk of non-vertebral fracture and DVT/PE, or those with risk factors for breast cancer.

While this study provides important insight into the differences between patients who initiate raloxifene and bisphosphonates for the prevention and treatment of osteoporosis, a few limitations should be noted. First, as with all claims data, available information is limited and relies on the coding decisions, for billing purposes, of a variety of professionals [22]. As such, the potential for coding errors or omissions should be recognized. Furthermore, it is not possible to directly assess the primary medical condition (e.g., osteoporosis) prompting a particular prescription, much less the true motivation (in the mind of the prescriber) leading to selection of one agent over another.

It should also be noted that the Medicare patients included in this study are those with supplemental insurance provided by employers. Because the characteristics and experiences of patients with Medicare coverage alone may differ from patients with supplemental insurance, the extent to which our results are generalizable to the entire Medicare population is unclear. Finally, medication formulary status was unavailable. Therefore, it is unclear how formulary placement may have impacted treatment patterns.

## Conclusion

The findings of this study suggest that patients initiating raloxifene for the prevention and treatment of osteoporosis differ from those initiating bisphosphonates. Patients initiating raloxifene tend to be younger, have better overall health status and may have fewer risk factors for new osteoporotic fractures than patients initiating bisphosphonates. Differences in the clinical profiles of these medications may impact prescribing decisions. Investigators using observational data to make comparisons of treatment outcomes associated with these medications should take these important differences in patient characteristics into consideration.

## Competing interests

Funding for this study was provided by Eli Lilly and Company. The following individuals are employees and stockholders of Eli Lilly and Company: Shonda A. Foster, Eric S. Meadows, Joseph A. Johnston and Gerhardt M. Pohl. The following individuals are of Thomson Medstat. Thomson Medstat received funding from Eli Lilly and Company to conduct the study: Kathleen A. Foley, Sara Wang and Stacey R. Long.

## Authors' contributions

SF conceived of the study, and participated in its design and coordination and helped to draft the manuscript. KF participated in the design and coordination of the study and helped to draft the manuscript. EM participated in the design of the study and helped to draft the manuscript. JJ participated in the design of the study and helped to draft the manuscript. SW participated in the design of the study and performed the statistical analysis. GP participated in the design of the study and provided guidance on the statistical analysis as well as drafting of the manuscript. SL participated in the design of the study. All authors read and approved the final manuscript.

## Pre-publication history

The pre-publication history for this paper can be accessed here:



## Supplementary Material

Additional file 1 **Codes for identifying confounding conditions and screening tests.** The appendix contains additional information on all codes (e.g., ICD-9, CPT) used to identify confounding conditions and screening tests in the study. Click here for file

Additional file 2 **Attrition of study sample following application of exclusion criteria.** The appendix contains detailed information on attrition of the study sample after application of the exclusion criteria. Click here for file
