# Supplementary material for: Characteristics of patients initiating raloxifene compared to those initiating bisphosphonates
Source: BMC Womens Health. 2008 Dec 23;8:24. doi: 10.1186/1472-6874-8-24 (PMC2642779; doi:10.1186/1472-6874-8-24)
Supplement: Additional file 1 — Codes for identifying confounding conditions and screening tests. The appendix contains additional information on all codes (e.g., ICD-9, CPT) used to identify confounding conditions and screening tests in the study. [file 1472-6874-8-24-S1.doc]

**Appendix A: Codes for identifying confounding conditions and screening tests**

| **Variable** | **Codes** |
| --- | --- |
| **Mammogram** | ICD-9: 87.36, 87.37, V76.11, V76.12  CPT: 76090-76092  UB-92 Rev codes: 0401, 0403 |
| **BMD Test** | CPT: 76070, 76071, 76075,76076, 76078, 76499, 76977, 76999, 78350, 78351 |
| **Esophagitis** | ICD-9: 530.10-530.12, 530.19 |
| **Dysphagia** | ICD-9: 787.2x |
| **Reflux** | ICD-9: 530.81 |
| **Gastric ulcer** | ICD-9: 531.00, 531.01, 531.10, 531.11, 531.20, 531.21, 531.30, 531.31, 531.40, 531.41, 531.50, 531.51, 531.60, 531.61, 531.70, 531.71, 531.90, 531.91 |
| **Peptic ulcer** | ICD-9: 533.00, 533.01, 533.10, 533.11, 533.20, 533.21, 533.30, 533.31, 533.40, 533.41, 533.50, 533.51, 533.60, 533.61, 533.70, 533.71, 533.90, 533.91 |
| **Gastric ulcer** | ICD-9: 534.00, 534.01, 534.10, 534.11, 534.20, 534.21, 534.30, 534.31, 534.40, 534.41, 534.50, 534.51, 534.60, 534.61, 534.70, 534.71, 534.90, 534.91 |
| **Gastritis** | ICD-9: 535.00, 535.01, 535.30, 535.31, 535.40, 535.41, 535.50, 535.51 |
| **DVT/PE** | ICD-9: 453.40-453.42, 453.8 (prior to 10/1/2004) and 415.1x |
| **Fractures** | ICD-9: 79.0-79.3, 79.6, 733.1x, 805.xx-806.xx, 807.0x-807.3x, 808.xx-815.xx, 818.xx-825.xx, 827.xx, 828.xx |
| **Breast Cancer** | ICD-9 : 174.x, 175.x, 238.3, 239.3  ICD-9 procedures : 85.22, 85.23, 85.4x  CPT: 19160, 19162, 19180, 19200,19220, 19240 |
| **Endocrine disease** | ICD-9: 250-259 |
| **HIV** | ICD-9: 042, 043, 044, V08 |
| **Liver disease** | ICD-9: 070, 570-573 |
| **Bone Cancer** | ICD-9: 170.0 – 170.9 |
| **Other Cancer** | ICD-9: 140.x – 208.x, 230.x-239.x, except 170.x (bone cancer), 174.x175.x (breast cancer) and 172-173.x (melanoma) |
| **Alcoholism** | ICD-9: 303.x |
| **Osteodystrophy** | ICD-9: 756.5 |
| **Nephritis** | ICD-9: 580.xx-589.9 |
| **Rheumatoid Arthritis** | ICD-9: 714.0x, 714.1x, 714.2x, 714.3x |
| **Cardiovascular Disease** | ICD-9: 410.xx-414.xx |
| **Thyroid Disease** | ICD-9: 240-246.9 |
| **Metabolic Disorders** | ICD-9: 270-278.8 |
| **Osteoporosis** | ICD-9: 733.00-733.03, 733.09 |
