# Supplementary material for: Characteristics of patients initiating raloxifene compared to those initiating bisphosphonates
Source: BMC Womens Health. 2008 Dec 23;8:24. doi: 10.1186/1472-6874-8-24 (PMC2642779; doi:10.1186/1472-6874-8-24)
Supplement: Additional file 2 — Attrition of study sample following application of exclusion criteria. The appendix contains detailed information on attrition of the study sample after application of the exclusion criteria. [file 1472-6874-8-24-S2.doc]

**Appendix B: Attrition of study sample following application of exclusion criteria**

|  | **Commercial/Medicare** | | **Medicaid** | |
| --- | --- | --- | --- | --- |
|  | **Raloxifene** | **Bisphosphonates** | **Raloxifene** | **Bisphosphonates** |
| At least 1 claim for index drug in the index period | 102,750 | 356,145 | 42,770 | 184,193 |
| Exclusion Criteria |  |  |  |  |
| Age <45 | 939 | 7,474 | 655 | 7,045 |
| Prior use of osteoporosis medications | 44,792 | 116,720 | 26,571 | 86,810 |
| Not continuously enrolled for at least 18 months | 38,155 | 141,066 | 3,710 | 19,170 |
| Index drug days supply ≤ 0 days | 9 | 46 | 0 | 2 |
| Index drug days supply > 180 days | 1 | 36 | 0 | 8 |
| Prescription coverage not continuous | 457 | 1,399 | 0 | 0 |
| Men | 206 | 8,338 | 121 | 10,384 |
| Paget’s Disease | 208 | 1,175 | 209 | 893 |
| Total number of eligible patients | 17,983 | 79,891 | 11,504 | 59,881 |
